# Integrating knowledge on green infrastructure, health and well-being in ageing populations: Principles for research and practice

**DOI:** 10.1007/s13280-022-01765-5

**Published:** 2022-08-06

**Authors:** Matthew Dennis, Adam Barker, Jamie Anderson, Jenna C. Ashton, Gina Cavan, Penny A. Cook, David French, Anna Gilchrist, Philip James, Christopher Phillipson, Konstantinos Tzoulas, C. Philip Wheater, Ada Wossink, Sarah Lindley

**Affiliations:** 1 School of Environment Education and Development, University of Manchester, Arthur Lewis Building, Manchester, M13 9PL UK; 2 School of Environment Education and Development, Manchester Urban Institute, University of Manchester, Arthur Lewis Building, Manchester, M13 9PL UK; 3 Institute for Cultural Practices, University of Manchester, Manchester, M13 9PL UK; 4 Department of Natural Sciences, Faculty of Science and Engineering, Manchester Metropolitan University, Chester Street, Manchester, M1 5GD UK; 5 School of Health and Society, University of Salford, Allerton Building, Salford, M6 6PU UK; 6 Division of Psychology and Mental Health, Manchester Centre for Health Psychology, University of Manchester, Manchester, M13 9PL UK; 7 School of Social Sciences, Manchester Institute for Collaborative Research on Ageing, University of Manchester, Manchester, M13 9PL UK; 8 Department of Economics, School of Social Sciences, The University of Manchester, Manchester, M13 9PL UK

**Keywords:** Ageing, Health and well-being, Knowledge co-production, Social-ecological systems, Trans-disciplinary, Urban green infrastructure

## Abstract

Ageing and urbanisation pose significant challenges for public health and urban planning. Ageing populations are at particular risk from hazards arising from urbanisation processes, some of which are in turn exacerbated by climate change. One approach for mitigating the negative effects of urbanisation on ageing populations is the leveraging of the beneficial effects of urban green infrastructure as a public health intervention in the planning process. We assessed the potential of available theoretical frameworks to provide the context for such leverage. This involved active engagement with academics and practitioners specialising in ageing, green infrastructure and health and well-being through a knowledge-brokering approach. We concluded that an integrated and comprehensive framework on the socio-cultural-ecological determinants of health is lacking. To address this, we present a set of principles for overcoming challenges to knowledge integration when working at the intersection of green infrastructure, ageing, health and well-being. Our findings—and the co-production process used to generate them—have wider significance for trans-disciplinary research into the benefits of the natural environment to human health and well-being as well as other complex and interconnected topics associated with global grand challenges.

**Supplementary Information:**

The online version contains supplementary material available at 10.1007/s13280-022-01765-5.

## Introduction

Population ageing and urbanisation are two of the most important trends of the 21st Century with respect to public health and environmental planning. Demographic trajectories suggest that the current global population over 60 years of age, 962 million, will more than double by 2050 to 2.1 billion (UN 2018). By the same year, 66% of people worldwide are expected to live in urban areas. Although an ageing urbanising society might reflect the culmination of human societal achievements (van Hoof et al. 2018), it also brings with it challenges related to good health and well-being, for example, staying active whilst ageing (Plouffe and Kalache 2010), and providing socially inclusive environments for e.g. older adults (WHO 2007; Kabisch et al. 2017). Studies have shown that components of the urban environment contribute to the prevalence of leading global causes of death, including heart disease, cancer and respiratory disorders. For example, this can be through promotion of more sedentary lifestyles (Lee et al. 2012; Frank et al. 2019) and increased environmental stressors which are likely to affect older groups more than younger ones (Marselle et al. 2021).

These challenges highlight the need to understand how our urban environments can support ageing populations but in a sustainable way that promotes better health and well-being in later life. The demographic shift towards the migration of people, of all ages, to urban centres is a primary driver behind multi-national efforts to ease the global health burden. The ability of urban green space to facilitate health-promoting factors such as increased physical activity, links to cultural and biological heritage, improved social ties, physical and mental stress reduction and connectedness to nature has been asserted by the World Health Organization (WHO 2017). Evidence highlights the links between urban greenery and increased physical activity, quality of life and better health in ageing populations (Astell-Burt et al. 2014; Gong et al. 2014; Dennis et al. 2020). Therefore, the extent and quality of urban green space becomes critical for addressing a range of health and age-related planning goals.

### Articulating determinants of health for urban residents

Despite their importance, the interactions between ecological, social and cultural determinants of health remain poorly understood. Associated dynamics present a ‘wicked problem’ (Head and Alford 2015). For example, socio-cultural and demographic factors influence how much particular urban green spaces are used, who uses them and for what sorts of uses (Wolch et al. 2014). The interplay between factors that mediate both participation with, and the health benefits arising from, the natural environment is therefore highly complex. The need to address such complexity becomes acutely relevant when considered in light of the growing likelihood of climate-related hazards and the changing nature of vulnerabilities, particularly in urban areas.

In order to meet this growing agenda, models of well-being focussing on a wide range of determinants have been developed that go beyond traditional medical models of health. The former seek to promote an understanding of human well-being as an emergent state underpinned by coupled social and natural processes. This move towards understanding health in terms of socio-environmental holism has transformed the public health agenda, with seminal contributions such as Dahlgren and Whitehead’s (1991) Rainbow Model describing key determinants of health (subsequently modified in Barton and Grant’s 2006 health map for local human habitats) being key to understanding why inequalities in health persist. Such models represent an advance in terms of recognising the plurality of factors influencing human health and the connections, pathways and “knock-on” effects between the natural, built, social and economic environments. A recognition of the overarching influence of the natural environment on human well-being is a significant development towards the integration of social and ecological factors impacting health.

At the same time, whereas socio-environmental characteristics are acknowledged in public health models, their direct and indirect influence remains largely unarticulated. For example, socially oriented characteristics (such as community cohesion) are often cited as outcomes of human–nature interactions in conceptual models (Hartig et al. 2014; James et al. 2015; Jennings et al. 2016; Jennings and Bamkole 2019). However, there are fewer models that attempt to include socio-economic and demographic status as motivating factors (see Lachowycz and Jones 2013 though for a promising example). This appears to be the case for two main reasons: firstly, frameworks are typically informed by reviews of the literature rather than empirical, deliberative approaches aimed at documenting values and perspectives. Secondly, common terms of reference that can describe key concepts (such as “nature”, “value” or “well-being”) whilst capturing the inherent plurality of meaning in their usage are absent.

In order that social-ecological models of health can be effectively operationalized, we propose several requirements for trans-disciplinary research. These are, (i) the need for a holistic approach to defining health and well-being, (ii) an acknowledgement of ageing and the life-course when researching and planning nature-based interventions, (iii) understanding that socio-cultural factors moderate the relationship between people and the natural environment, and (iv) a recognition of the plurality of values and methods of valuation of nature as key considerations in the planning process. Although nested social-ecological models achieve a certain holism by identifying a range of determinants of health and well-being which are influenced by the natural environment, we argue that, in order to meet the aforementioned challenges, a fuller understanding and integration of the interplay between such determinants is required. Towards such an integration, we began with a core conceptualisation of urban nature and human health and well-being that draws on a green infrastructure (GI) model (Benedict and McMahon 2012) which has been widely adopted due to the demonstrable environmental benefits it can deliver (Hansen and Pauleit 2014).

### Green infrastructure: A nested approach for understanding public health outcomes

We argue that a GI approach is suitable for framing the integration of socio-environmental determinants of health and well-being in ageing populations. We do so for three reasons. Firstly, it encompasses aspects of the natural, social and institutional contexts for understanding health and well-being influences. Secondly, the notion of GI has been identified as an effective approach mainstreamed by local, national and international authorities (Hansen and Pauleit 2014). The concept has proven useful for strategic planning at a variety of scales (Weber and Allen 2010), and has demonstrated efficacy in mapping the provision of ecosystem benefits (Haase et al. 2014; Coutts and Hahn 2015), and capturing the multi-functionality of urban green space (Hansen and Pauleit 2014). Thirdly, a GI approach is designed to bring about the integration of trans-disciplinary perspectives (Lovell and Taylor 2013) in order to meet social, economic and ecological goals, and challenges associated with land-use planning (Weber and Wolf 2000; Weber et al. 2006; Benedict and McMahon 2012). The full promise of a GI approach is realized, therefore, through both the spatial-ecological benefits it may deliver as well as through the promotion of participatory and trans-disciplinary approaches to decision-making. However, comprehensive conceptual models which achieve the integration of GI, ageing, health and well-being are currently lacking. The challenges of overcoming disciplinary barriers, acknowledging the needs of different socio-demographic groups and the integration of the plurality of values within a GI approach require the assimilation and integration of a range of perspectives from both research and practice. In order to address this need, in this paper, we focus on three main questions:

1. What considerations and perspectives exist for understanding the range of health and well-being benefits and the plurality of values associated with urban green infrastructure for ageing populations?
2. How well are they represented in current frameworks and conceptualisations?
3. What are the challenges to leveraging GI in research and practice related to ageing, health and well-being?

Although, of particular relevance for those working in the same field of enquiry, we anticipate that the processes developed will have wider significance for teams of researchers and partner organisations working on similarly complex topics.

## Materials and methods

**Fig. 1:**
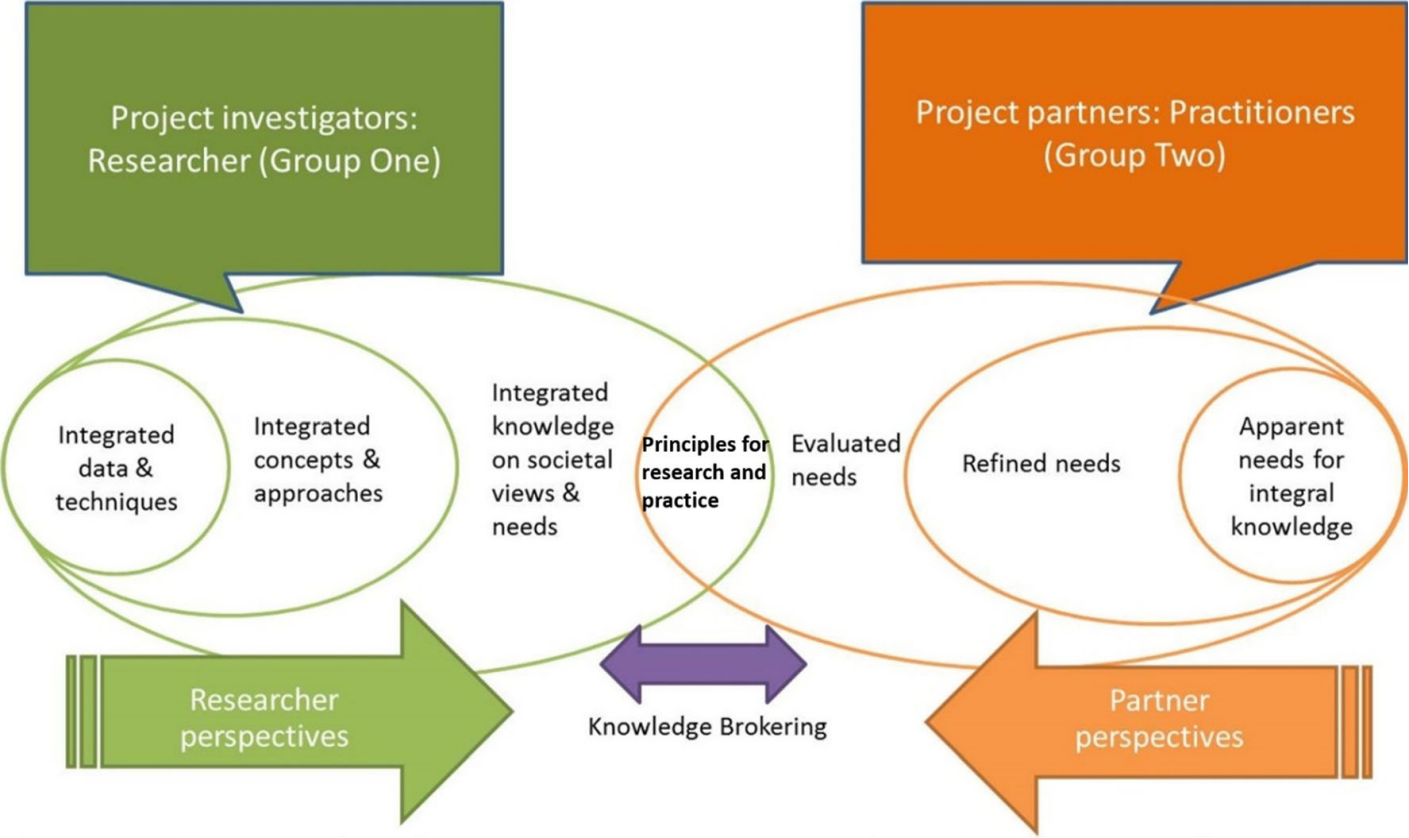
Knowledge-brokering format adopting in the study. Adapted from (Assmuth and Lyytimaki 2015)

We followed a knowledge-brokering process (Holzmann 2013) which can take many forms and apply to multiple contexts from research, practice, policy, commerce and the public domain. Knowledge-brokering methods have evolved primarily in response to both the increasingly complex nature of social-ecological challenges, or “wicked problems”, as well as to the prominence of knowledge-intensive areas of research which seek to address these challenges (Michaels 2009). They are therefore particularly suitable for exploring perspectives, theory and practice at the intersection of GI, ageing and health and well-being. Although other approaches exist which draw on stakeholder consultation in the decision-making process (such as multi-criteria mapping and Environmental Impact Assessments), the knowledge-brokering approach has proven effective in addressing broad and complex social-ecological problems (Dagenais et al. 2015; Assmuth and Lyytimaki 2015). Multi-criteria-based and other similar decision-making approaches have also proved to be effective at exploring health-related behavioural change (Kelly and Barker 2016). However, our aim was to establish a broader set of principles of working as a more fundamental and transferable basis for research and practice at the intersection of environment, ageing, health and well-being. It was not our goal to explore specific health-related behaviours but rather to provide a vehicle for leveraging GI as a public health intervention. In order to meet these research aims, in this study, we focussed on the interface and exchange of knowledge between experts from research and practice. We drew on a model developed by Assmuth and Lyytimaki (2015), which aims to co-create new knowledge through the reflective dialogue between, and integration of, research-driven and solution-driven perspectives. The format of this knowledge co-creation process is visualized in Fig. 1.

The knowledge-brokering process was designed to meet two research aims: firstly, arriving at an informed analysis of the current state-of-the-art in relation to concepts, conceptual models and other framings of GI, ageing, health and well-being; secondly, the identification of terms of reference and conceptual stances related to the field of enquiry and their subsequent integration. This process involved the participation of two key groups representing different forms of knowledge on the topics of interest. Group One (thirteen members) was made up of disciplinary (academic) specialists in the fields of ecology, planning, geography, economics, environmental ethics, philosophy, well-being, psychology, arts and heritage, gerontology and public health. Group Two (twelve members) consisted of practitioners with specialisms in the areas of public health, local environment, forestry, ecology, urban planning, nature conservation, public engagement, local governance and age-friendly cities. The purpose of Group Two was to provide a practitioner perspective and practice-based knowledge and experience to the brokering process. The geographical focus of the research was Greater Manchester (GM) UK, a city region of 2.8 million inhabitants spanning a large range of socio-demographic and ecological (i.e. urban to rural) contexts with a long history of acknowledging the role of GI in public health (Gilmore and Doyle 2019). The research involved experts (academics) from all three of the major universities in the city region (University of Manchester, Manchester Metropolitan University and Salford University) and practitioners (members of Group Two) likewise came from GM organisations (representing both the Greater Manchester Combined Authority and other Local Authorities within GM) with extensive local knowledge.

**Fig. 2:**
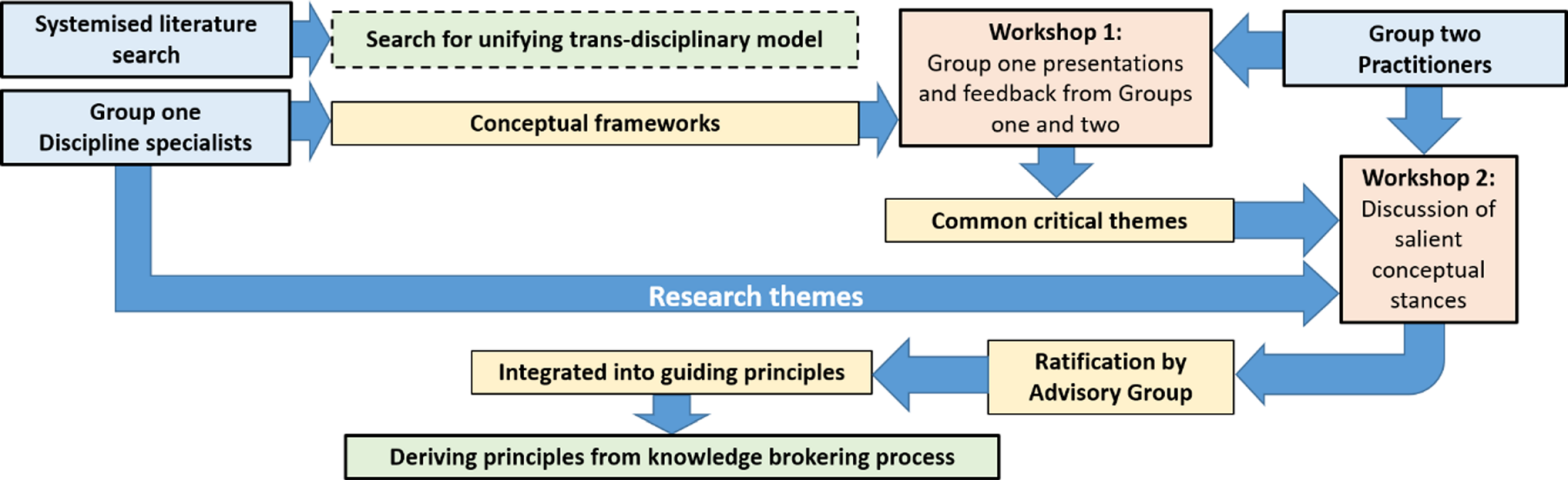
Flowchart of the methodological approach leading to the derivation of guiding principles

The knowledge-brokering process consisted of two workshops that took place in September 2016 and April 2017 and attended by members of both participant groups and follow up meetings with the advisory group for the research project. The latter comprised expert practitioners and academics selected for their knowledge and expertise in the areas of public health, ageing and GI. The role of the advisory group was to provide direction and guidance to Group One in delivering the wider project aims. The inclusion of members from Group Two within the advisory group provided a necessary degree of continuity and quality control throughout the research process. Briefly, Workshop One was aimed at evaluating the extent to which existing frameworks integrate and provide useful linkages between concepts relevant to GI, ageing, health and well-being. Workshop Two was designed to explore the diversity of perspectives from theory (Group One) and practice (Group Two) in order to establish a common understanding of these key concepts. Finally, knowledge gained through the evaluation of existing frameworks and the establishing of a common understanding of concepts at the intersection of GI, ageing, health and well-being was integrated into a set of principles of working for leveraging GI within public health research and practice. This process and the resulting principles were ratified through consultation with the advisory group (see Fig. 2 for an overview of the process). Workshops, and therefore the results in this paper, were informed by literature reviews conducted in 2016 and therefore will not reflect work published after this date.

### Identification and evaluation of contemporary approaches to conceptualizing the inter-relationships between GI, ageing, health and well-being (Workshop One)

For Workshop One, a preparatory piece of work was carried out by members of Group One. This consisted of an elicitation of conceptual frameworks representing the state-of-the-art of research on GI and human well-being. Group One members were asked to identify the prominent frameworks from their respective disciplines (see Table 2) relevant to the intersection of GI, ageing, health and well-being. In addition, a systematic literature search was carried out by a member of Group One (in August 2016) to identify existing conceptual frameworks which combined the themes at the centre of this research, namely GI, health, well-being and ageing (see Supplementary Materials for details of search terms used). Subsequently, members of Groups One and Two were invited to participate in a workshop designed to facilitate a critical evaluation of identified frameworks. The key elements of identified frameworks relevant to GI, ageing, health and well-being were summarised in an academic poster format for the benefit of participants. A poster review session captured initial commentary through the use of sticky notes appended to each in a carousel format. A subsequent discussion took place to clarify points raised in this initial process with notes taken by a member of Group One and agreed upon by participants.

### Establishing a common terminology for understanding the links between green infrastructure and health and well-being (Workshop Two)

The development of shared concepts is a key element of knowledge brokering, necessary in order to establish a common lexicon and subsequent dissemination of information and practices (Assmuth and Lyytimaki 2015). Workshop Two explored the challenges faced in seeking a common use of terminology and attempting to integrate complex themes related to ageing, human health and well-being and the influence of GI. Here, members of Group One again provided the input for discussion. Specifically, members of Group One were asked to provide briefing documents detailing the theoretical perspectives and definitions that their respective disciplines employ to understand the key areas of GI, ageing, health and well-being in urban areas. These were identified, discussed and assigned to individual experts at a pre-workshop meeting attended by Group One. Briefing documents were then provided to members of Group Two in advance of Workshop Two and as oral presentations on the day. An open discussion of these definitions and concepts was facilitated within the workshop environment in order to (a) document divergent views, (b) establish a common understanding of terminology and (c) explore challenges and opportunities for integrating key concepts relevant to GI, ageing, health and well-being. Outputs from this discussion were recorded by note-takers and collated with those acquired through workshop one. All outputs were subsequently coded into common themes by a member of Group One and ratified by the wider team. Members of Group One then evaluated the workshop outcomes to highlight areas where existing frameworks and conceptual stances failed to effectively integrate the multiple perspectives and knowledge articulated by participants in the co-production process. Finally, the diverse views captured from Group One and Group Two within the workshop environment were synthesized though the creation of a set of guiding principles, aimed at enhancing health and well-being in ageing populations through a GI approach. The principles were the result of condensing the two strands of the knowledge-brokering process, namely: the shortcomings identified through the critique of existing frameworks (Workshop One) and the application of expert perspectives on concepts related to GI, ageing, health and well-being (Workshop Two) to these shortcomings. These principles were initially drawn-up by members of Group One and were presented to the project Advisory Board at a further meeting where members had the opportunity to provide feedback and shape the final version of the proposed principles. A summary of how the various stages of the brokering process linked to produce these principles is given in Fig. 2.

## Results

### Consultation and literature search: Identifying conceptual frameworks

Consultation with Group One participants led to the identification of seven conceptual frameworks. Table 1 presents basic details of identified frameworks for consideration, detailing their purpose, key concepts and the primary academic discipline of origin. The systematic literature search did not identify any existing frameworks that deal with the full range of disciplines and themes at the centre of the research activity, returning no articles for consideration.

**Table 1:** Framework overview

Name of framework	Source	Purpose	Key concepts and approach	Workshop One Commentary
Ecosystem services cascade	Haines-Young and Potschin (2010)	Clarify the links and terminology surrounding ecosystem processes and the benefits that humans receive/perceive Address the issue of varying and conflicting typologies and interpretations around ecosystem service provision and value	The cascade represents a “production chain” from ecological process to end-user benefit Services and associated values are context-specific Framed within the Ecosystem Approach and social-ecological systems model	• Leans on ecosystem services as a proxy for well-being • Benefits derived from ecosystem services quantified in anthropocentric, utilitarian terms with an economic emphasis • Absence of direct links to human health
Fragments, Functions, Flows & Urban Ecosystem Services (F3UES)	Biodiversity and Ecosystem Services Sustainability (Grafius et al. 2018)	Explore how the biodiversity of towns and cities contributes to the provision of Ecosystem Services (ES), and hence, human well-being	Stocks and flows: Biodiversity is seen as a ‘stock’ (similar to natural capital), from which the ‘flows’ of ES are delivered Relationship between GI and ES: ES in urban areas are often framed in the context of GI and that ES research needs to be consistent with this	• Strong spatial aspect grounded in ecosystem services • No emphasis on valuation • Well-developed consideration of the influence of scale on nature’s benefits to people
Intergovernmental Science-Policy Platform on Biodiversity and Ecosystem Services (IPBES)	IPBES (Diaz et al. 2015)	To frame IPBES activities around its long-term goal of conservation and sustainable use of biodiversity, long-term human well-being and sustainable development to: Encourage new knowledge creation Review/assess existing knowledge Support policy-making Build science-policy-practice capacity	Mutual recognition and enrichment among different disciplines and knowledge systems Recognises anthropocentric aspects of assessment of values, open to pluralistic and non-monetary framing Considers roles of time and space and recognises scale dependencies Use of multiple terminologies and open to context dependent alternatives	• Although grounded in anthropocentric concepts of nature conservation, also the only framework to consider relational value as a progression beyond polarised utilitarian versus intrinsic views on the value of nature • Only framework to consider temporal scales in the context of environmental processes and human health
Green Infrastructure, Ecosystem and Human health (GIEH)	(Tzoulas et al. 2007)	Encourage the integration of information among and between disciplines Review, identify and categorise different academic traditions, research methods, specialised language, and theories	Green infrastructure (all green spaces and their physical and functional interconnections) Human health (dynamic state of physical, psychological and social well-being) Human well-being (defined through socio-economic and psychological factors, including connectedness to nature) Ecosystem health (dynamic and resilient to stress, maintaining organisation, productivity, autonomy)	• No emphasis on valuation • Only framework to offer direct links between ecosystems processes and human health • Acknowledgement of influence of different scales of resolution in the assessment of ecological and health indicators • No consideration given to concepts or methods relevant to valuation
Conceptual framework for Multi-functionality in Green Infrastructure Planning for urban areas (MGIP)	Hansen and Pauleit (2014)	Integrate dual-management of co-existing Green Infrastructure, its multi-functionality and Ecosystem Services (ES)	Ecological—assessing current provision of GI (spatial elements and structures) through appropriate ecological indicators Social element addresses demand for services as a key planning consideration Valuation: identification of GI integrity, ES hotspots, trade-offs, supply–demand balance and stakeholder preferences Discrete ecosystem services influence people at different scales (e.g. local, distant, cross-scale, uni-directional)	• Operational focus with some acknowledgement on the role of societal choice and valuation in green space planning • Present examples of a participatory approach though with no clear guidance on its implementation • Strong emphasis on multi-functionality and the effect of local, distant and uni-directional scales of influence in well-being benefits from green space
Valuing Nature Network (VNN) exploratory work	https://valuing-nature.net/	Develop an improved understanding and representation of the complexities which surround the role of the natural environment in both valuation and decision-making processes	Establishing robust measures and methods of valuation of nature (monetary and non-monetary) Consideration of the economic, societal and cultural value of ecosystem services Preferences as a mediating stage influencing realistic valuation of ES	• Driven by a focus on evaluating the possibility of valuing nature in market-based terms • The framework acknowledges the importance of shared values but an emphasis on monetary valuation may create barriers for its application in the context of participatory approaches
Green Space and Health: a conceptual framework (GSaH)	Lachowycz and Jones (2013)	Identify the moderating and mediating factors which affect the relationship between green space and health benefits	Demographic, socio-economic and environmental factors are considered in assessment of benefits of exposure to, and use of, green space. Understanding the mechanisms of moderation is key to interpreting results of research	• No emphasis on valuation • Of all the frameworks considered, the clearest treatment of the mediation of health outcomes by socio-demographic factors • Poor representation of ecological or scale-related factors with a bearing on health

### Summary of findings from the framework evaluation (Workshop One)

The findings from the workshop revealed that a full integration of the dominant themes of GI, health and well-being, ageing and valuation (as identified in the introduction to this paper) is currently lacking in the leading scientific literature on the topic. Frameworks issuing from the natural sciences (e.g. Ecosystem Services Cascade, F3UES, MGIP) represent a principal driving force in the conceptualisation of benefits to human health and well-being stemming from the natural environment. Of note was the fact that public health research has largely failed to put forward conceptual models of working, despite this field of enquiry having generated much evidence on associations between GI and health. Another omission in attempts to provide a conceptual road map in social-ecological approaches to health and well-being concerns guidance on relational and participatory routes to valuation (with the exception of the VNN and IPBES frameworks). Similarly, there was a lack of explicit reference to cultural heritage value in assessments of GI, human health and well-being in all frameworks considered (though IPBES provide a potential framework to acknowledge such motivations through its emphasis on relational value).

### Identification of academic and practitioner perspectives towards the integration of key concepts and theory (Workshop Two)

The key concepts and of theoretical viewpoints associated with Group One research specialisms, and presented to members of Group Two, are listed in Table 2 under the six academic research specialisms. Table 2 contains both underpinning concepts and academic standpoints, according to the contributions from the different academic teams, which in some cases also included an emphasis on particular approaches and methods.

**Table 2:** Key concepts and theoretical viewpoints related to green infrastructure, ageing and well-being from Group One disciplinary perspectives

Research specialism	Disciplinary concepts and principles relevant to the intersection of GI, ageing, health and well-being
Green Infrastructure	1. *Multi-functionality* (ecological, social and economic functions considered in parallel); 2. *Physical and functional connectivity* (linking people, habitats, function and species); 3. *Social inclusion* (societal needs are central to the planning process); 4. Trans-disciplinarity (drawing on knowledge from relevant disciplines and stakeholders) 5. A green infrastructure approach acknowledges the *influence of scale* in developing nature-based solutions to social-ecological challenges
Health	1. *Rise of chronic conditions* in the industrialized world (pathogenic model); 2. *Integrated social-ecological models of health* Individual health outcomes as the results of interacting biological, social and environmental factors;
Well-being	1. *Subjective state theory*/hedonism (feeling good): (after e.g. Epicurus; Jeremy Bentham), Well-being consists in having certain subjective psychological states 2. *Preference satisfaction theory* Well-being consists in the satisfaction of personal preferences, the stronger the preferences, the greater the well-being; 3. *Objective list/state theory* Well-being consists in the realisation or capacity to realise of certain objective goods or states such as particular forms of personal relation, physical health, autonomy, knowledge of the world, aesthetic experience, accomplishment and achievement, sensual pleasures, a well-constituted relation with the non-human world, and so on 4. *Capabilities approach* Well-being is defined in terms of in terms of *capabilities* to achieve central human *functionings* *Functionings* ‘the various things a person may value doing or being’—what people are able to be and do *Capabilities* ‘substantive freedoms to achieve alternative functioning combinations’ (Sen 1993 p.75)
Ageing	1. Acknowledgement of *diversity* within the ageing population; increase in demand for care with increasingly ageing populations; 2. There exists a need to address *needs of black and minority ethnic group communities*; 3. The central *role of the neighbourhood* in quality of life in older age; 4. *Environmental volunteering* may carry particular health benefits in later life 5. *Co-research* with older adult participants
Arts and Heritage	1. *Cultural heritage* Ways of living passed down through generations including *Intangible Cultural Heritage* such as oral traditions, performing arts, social practices, knowledge of the natural world, traditional craftsmanship 2. *Safeguarding* measures (e.g. as adopted by UNESCO) aim to promote and protect the viability of intangible cultural heritage, prioritizing opportunities for women’s empowerment 3. *Creative practice* as a method of participatory engagement
Value and valuation	1. *Monetary valuation* Assumes that monetary values can be assigned to features of the natural environment to facilitate their inclusion in market-based decisions, thereby highlighting their worth 2. *Participatory methods of valuation* (e.g. deliberative/democratic approaches): attempt to capture a range of values and perspectives hidden from monetary assessments

The discussion based on concepts, standpoints and theories presented by members of Group One brought together a broad range of academic and practitioner perspectives for integration. Key discussion points related to knowledge, emphasis and challenges associated with research themes are summarised in Table 3.

**Table 3:** Summary of perspectives documented through Workshop Two. Group One (academics) comments in normal font, Group Two (practitioners) comments in italics

Research theme	Workshop discussion points and perspectives
Green infrastructure	• Inconsistency in the monitoring and expressing of quality and value of green infrastructure assets is an obstacle to effective planning and evidence building • *There exists a need to consider both biological and social quality and provision in green infrastructure planning and research*
Health	• Measures of health need to be carefully considered in light of socio-economic contexts and quality/availability of data • *Behaviour, such as lifestyle and physical activity, is a key consideration in public health*
Well-being	• A key challenge lies in measuring the multi-dimensional value of components which contribute to well-being • The benefits of the natural environment on individual well-being are shaped by socio-cultural factors • *This makes qualities of the natural environment difficult to translate into meaningful estimates of well-being benefits*
Ageing	• Conceptualization of older generation can be misleading as overarching definitions conceal the diversity present in ageing populations • Narrow approaches to interpreting and defining elements of green infrastructure can also present barriers to participation in nature-based activities • *Transitions within the life-course can be watershed moments for the individual’s relationship with the natural environment*
Heritage	• Capturing the value of heritage of all kinds remains a considerable challenge in social-ecological research and policy • *Emphasis should be placed on people and person-centred approaches as opposed to artefacts in explorations of culture and heritage* • *In practice tensions can arise between person-centred approaches to safeguarding and promoting heritage and culture, and asset management*
Value and valuation	• Monetary valuation cannot capture some dimensions of values such as meaning and sense of place • There is a danger of creating a binary classification of approaches to valuation (monetary versus non-monetary) and a need to recognise the plurality of value in order to create effective methods of accountability (e.g. in order to hold organisations environmentally accountable for their actions) • *Calculating value depends on the audience and should be tailored accordingly to the needs/perspective of the audience* • *Economic valuation can be important for decision-making and providing for sustainability in funded projects*

**Fig. 3:**
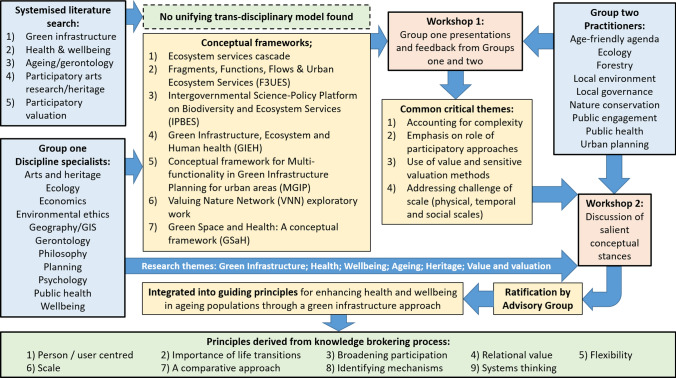
Flowchart of interim and final outcomes from the knowledge-brokering process

The knowledge generated through Workshops One and Two culminated in a set of nine principles for leveraging GI as a public health intervention. A summary of the interim and final outcomes from this work is given in Fig. 3.

### Description of the principles derived from the knowledge-brokering process (non-technical description in parentheses)

1. *Person/user-centred* (Involving people who are expected to benefit from the outputs of the research).
  An inclusive description of the relationship between individual health and well-being and GI should be sought to the furthest degree possible. This should be driven by knowledge on the particular perspectives, behaviours and needs of the individuals/communities under consideration.
2. *Importance of life transitions* (The need to consider the role of life transitions for understanding links between green infrastructure and health and well-being).
  Key to an age-related understanding of how people relate to and benefit from the natural environment (i.e. as embodied in GI) concerns how meaning is attached to the latter during important stages in the life-course, as can be observed through analysis of birth cohorts (see Phillipson 2017). There is an evidence to suggest (e.g. Smalldone et al. 2005; Bell et al. 2015) that nature-based activities often hold particular meaning and opportunity for enhancing well-being during transitional periods in the ageing process, such as leaving home, the birth of children or grand-children, retirement, or bereavement. An awareness of how well-being effects from the natural environment can be strongly mediated by stages in the life-course of the individual should therefore be considered where possible.
3. *Broadening participation* (Broadening participation in green and blue spaces associated with GI and in related decisions, such as its valuation)
  Approaches which seek to increase and diversify participation both in terms of use of GI and democratic methods of capturing value should be adopted. Participatory methods have been presented as vital components of resilient social-ecological systems by increasing the body of (local) knowledge available to decision makers as well as knowledge exchange and trust between stakeholders and practitioners (Walker et al. 2002; Olsson et al. 2004).
4. *Relational value* (Emphasising the importance of valuing the ways in which people relate to and are motivated to engage with the natural environment through urban GI).
  In reference to principles 1, 2 and 3, the adoption of the concept of relational value (in what ways people relate to and are motivated to engage with the natural environment) is paramount and provides a means of avoiding binary approaches to value (i.e. intrinsic vs utilitarian perspectives). For example, economic valuation of natural capital has proved not to be the panacea of environmental stewardship (Knights et al. 2013), with local to global ecosystems still subject to continued degradation and exploitation. Likewise, the intrinsic value of nature has yet to be effectively integrated into environmental policy. A relational approach avoids the pitfalls of adopting either stance by focussing on that which motivates individuals, communities and decision makers to place value on the elements of the natural environment in which they are stakeholders (Diaz et al. 2015). In turn this helps to understand how different individuals and groups can gain health and well-being benefits (or harms) in the future.
  This approach acknowledges the multiple ways in which individuals and communities connect with and notice their environment for health and well-being benefits such as is promoted, for example, through the Five Ways to Well-being (Farrier et al. 2019).
5. *Flexibility* (Research that is flexible and acknowledges the legitimacy of different perspectives and views).
  Acknowledging the plurality of perspectives that exist towards key concepts such as GI, health and value is vital in order to understand the preferences and well-being benefits expressed by diverse user groups present within social-ecological systems (Bennett et al. 2015). The complexity involved in converting benefits and qualities from one dimension (i.e. GI) to another (i.e. health and well-being) (Armitage et al. 2009) should take into account the multiple modes of expressing environmental quality, healthy outcomes and wider definitions of well-being. Deliberative approaches (such as Q-methodology and Deliberative Mapping see “Opportunities for further integration and effective implementation of the principles for leveraging GI as a public health intervention” section) provide promising starting points for articulating divergent perspectives, value plurality and reaching consensus on environmental quality and value-oriented decision-making (e.g. Forrester et al. 2015).
6. *Scale* (Considering spatial and temporal scales of social and GI-related processes and outcomes and how they influence research and practice).
  Where possible, all significant social, cultural and ecological factors influencing the relationship between the natural environment and health should be considered simultaneously at an appropriate scale. This process should acknowledge the influence of slower/faster variables operating at larger/smaller physical and temporal scales (Ernstson et al. 2010). For example, persistent socio-cultural characteristics of an area, or gradual economic decline, represent slow variables that may change over much longer periods of time than “faster” variables such as the modification of GI (whether increasing—“greening”—or decreasing i.e. development) or improving access to urban nature. GI interventions should therefore consider existing perspectives and preferences of particular communities.
7. *A comparative approach* (Working in a range of locations in order to produce evidence relevant to a variety of social and environmental contexts).
  A consideration of geographical patterns is key to understanding the provision of, and need for, GI benefits to an ageing population, including socio-cultural characteristics. Such an understanding is necessary for both a whole-system approach to understanding health and well-being and identifying local patterns of inequality for more focussed research or intervention-based work. The management of GI should consider the constraining influence of background social parameters which play a mediating role in peoples’ relationship with the environment. For example, it has been demonstrated that socio-economic and ethnic backgrounds can be mediators of engagement with the natural environment. Therefore, if GI interventions are to be effective, they should consider socio-cultural contexts and potential barriers to use.
8. *Identifying mechanisms* (Research that emphasises the range of pathways through which health and well-being are influenced by urban GI as we age).
  Appropriate hypotheses related to GI and human health and well-being drawn from, for example, psychology (e.g. attention restoration theory, ART), health-related behaviours (e.g. physical activity) or physiology (e.g. immune-regulatory pathways) should be researched and considered appropriately according to the nature of the work being undertaken. Underlying mechanisms (e.g. biological, psychological, cultural or behavioural, as appropriate) may inform research and interventions seeking to gain insight into or improve on the relationship between GI, human health and well-being. Similarly, the application of the principles to research may further inform current theory by providing new trans-disciplinary perspectives on the processes and values which determine health and well-being outcomes through GI and its attributes, such as biodiversity.
9. *Systems thinking* (An acknowledgement that GI operates as a system involving both people and the natural environment).
  Human-dominated landscapes should be viewed as coupled social-ecological systems with place-specific ecological, social and cultural characteristics and drivers interacting at a range of scales. Social-ecological systems are characterized by complexity and understanding is facilitated by drawing information from the social, ecological, historical, cultural and institutional aspects of a given system. Understanding the perspectives of actors from individuals to communities to organizations within social-ecological systems is likewise achieved by the same means (Berkes 2009). Diversity and connectedness of both socio-cultural and biological characteristics play a key role in managing the use, and resilience of system components (e.g. green spaces such as parks or riverine corridors) and the wider social-ecological system itself (Norberg et al. 2008; Biggs et al. 2012).

## Discussion

The knowledge-brokering process resulted in the capture of a broad range of perspectives (addressing research question one) which provided the basis of the development of a set of principles of working. Perspectives articulated in the workshop environment coalesced around two broad considerations, operating at different scales, for integrating knowledge at the intersection of GI, ageing, health and well-being.

Firstly, a strong emphasis on the importance of considering the user within their socio-cultural, economic and demographic (e.g. life-course) context was observed. Such characteristics were identified by practitioners and researchers alike as a strong influence on both the ability to engage with the natural environment and on the quality of the environment in which individuals live. This idea resonates in the wider literature on green space and health (Maas et al. 2009; Schipperijn et al. 2010) and highlights the need for context-specific evaluations of GI-ageing-health and well-being interactions. Broadening participation in decision-making, in order to capture unique viewpoints and motivations behind the use and valuation of GI, was highlighted. Linked to the importance of participation was a perceived need to capture value plurality in an effective and flexible way (Principle 5) and acknowledge value incommensurability where necessary (Table 3: “Value and valuation”). This was seen to be critical in addressing the needs of individuals and communities in the promotion and communication of health and well-being benefits, but comes with significant challenges (discussed in section “Appropriate use of value and sensitive valuation methods” with methodological opportunities presented in section “Opportunities for further integration and effective implementation of the principles for leveraging GI as a public health intervention”).

The need for a comparative approach was the second important theme identified, reflecting the influence of broader social, temporal and geographical processes which frame interactions between GI, ageing, health and well-being. For example, natural, socio-demographic and decision-making processes interact at different temporal scales which can lead to mis-matches in the provision of, and need for, urban GI (Borgström et al. 2006). Research reported elsewhere strongly supports this position (Buijs et al. 2016). Adopting a cross-scale perspective on GI, ageing, health and well-being is therefore essential in order to identify sources of inequality (at broader scales e.g. by comparing GI and socio-economic data at the neighbourhood or census level) as well as to capture motivations and preferences at the level of the individual to inform decision-making when GI interventions are planned.

The themes which stemmed from the knowledge-brokering process reflect the complexity of the nature of social-ecological systems themselves. In particular, the brokering process identified complex interactions influencing engagement with GI and that, in order to navigate such complexity, a systems approach (sensu Biggs et al. 2012) is necessary. For example, an individual’s ability to engage with GI may be conditioned by the socio-economic context (e.g. availability of green space, ability to travel; Maas et al. 2009) but also by demographic (e.g. age-related or cultural barriers; Schipperijn et al. 2010), physical (e.g. accessibility of, or appropriate facilities within, green spaces; Gong et al. 2016) or psychological (e.g. fear of crime: Hong et al. 2018) factors. In addition, individual contexts are constrained by larger-scale processes such as economic cycles, housing demand and environmental and political changes which may all affect different communities with different degrees of severity (Frank et al. 2017).

A focus on ageing as a factor moderating health and well-being influences from GI resulted in the surfacing of specifically demographic as well more generic concerns. For example, Principle 2 is concerned explicitly with stages within the life-course and how these affect positive (and negative) interactions with GI, whereas other principles (e.g. the need for a comparative approach: Principle 7) could be applied more generally outside the concept of ageing. Given the potential universality of some of the principles, the process undertaken underlines the importance of ageing as a key consideration in decision-making that can highlight issues pertinent to wider society. The emphasis placed on life transitions in our results asserts the ageing process as a dynamic and inevitable aspect of our relationship with GI that affects not only issues such as access and mobility but also relational value as different motivations and needs take precedence as we age (Douglas et al. 2017). Similarly, from the point of view of mechanisms (Principle 8), health and well-being benefits (and harms) may be conferred through different pathways (e.g. ART, physical activity, social and cultural ties) at different stages of the life-course. As a result, values towards GI may shift as one progresses into different stages of the life-course, underlining the need for a flexible approach (Principle 5) that acknowledges value plurality that is also temporally dynamic. Despite the promise of adopting ageing as a useful entry point to understanding GI and health, developing an approach to identify, integrate and manage the complex interactions implied presents a significant challenge.

### Challenges for knowledge integration and implementation of the principles

The process of developing the nine principles highlighted key challenges around knowledge and value integration that had thus far prevented the creation of a fully holistic and practicable framework for working at the intersection of GI, ageing, health and well-being (addressing research question three). Though the principles described are designed to overcome challenges to integration, there are key challenges relevant to implementation surfaced by the knowledge-brokering process that are important to acknowledge and navigate in order that the principles are effectively operationalized. Three key challenges to effective implementation were identified: (i) addressing complexity in the understanding of health and well-being and their determinants, (ii) understanding processes of valuation and, (iii) accounting for scale. In subsequent sections, we discuss these challenges, using the reviewed frameworks as points of reference (addressing research question two). We then suggest potential pathways to overcoming them.

#### Addressing complexity in the understanding of ageing, health and well-being

The co-production process highlighted that the level of clarity and integration of the themes of health and well-being with respect to ageing and GI were achieved to variable degrees by existing frameworks, with none of those considered achieving a full integration. This insight mirrors similar assertions in the literature on the need to navigate complexity in public health promotion in social-ecological systems (Bunch et al. 2011). For example, five of the seven frameworks placed strong emphasis on the concept of ecosystem services and employed the definition of the latter as a kind of proxy for human health and well-being based on the widely accepted definition that they represent “the benefits people obtain from ecosystems” (MEA 2005; (vi). This is the basic assumption of the F3UES, the MGIP framework, the Ecosystem Cascade model and the VNN framework. Therefore, these frameworks, reduced to assessments of ecosystem services flows and provision in their definition of well-being, appeared to be limited in their exploration of complexities related to health outcomes.

However, within the VNN framework, and especially within the IPBES approach, consideration is given to measures of human health and well-being which are conceptually discrete from the notion of ecosystem services. In their framework concerning GI and ecosystem and human health (GIEH), Tzoulas et al. (2007) draw on the concept of ecosystem services but emphasise rather the notion of ecosystem health and its influence on that of humans. In this sense, it is one of only two frameworks that succeed in providing a direct conceptual link between ecosystem processes and human health, offering concrete mechanisms for related health outcomes. This was a key priority (identifying mechanisms) highlighted in the co-production process as being critical to effective implementation. Likewise the GSaH (Lachowycz and Jones 2013) conceptualisation of human health outcomes related to green space provision focusses on characteristics of environmental features and how these may mechanistically affect interaction with green space. In this way, the GSaH framework provides the greatest detail of all the frameworks analysed in terms of articulating causal links between concepts. Moreover, by considering demographic variables (i.e. the social environment and related contexts), this latter framework is the sole one under consideration which makes explicit mention of age as a mediating factor in the relationship between human and environmental health. Though this would be considered a good example of thematic integration, according to the outcomes of our co-production process, these frameworks are purely analytical and offer little explanation as to the operationalization of such themes. Therefore, effective guidance on implementation remains a key omission in the literature on GI, ageing, health and well-being.

A key area of concern amongst practitioners (Group Two), for any framework attempting to leverage GI towards improved public health outcomes, related to the need to recognise the complexities and uncertainties present in the degree to which socio-cultural factors play a mediating role in health and healthy relationships with the natural environment. Inconsistencies were highlighted in modes of working and knowing between and within research and practice that can compromise effective implementation of research into health, well-being and GI. These inconsistencies related primarily to indicators and measures of well-being and quality of natural spaces, as well as evidence building and its documentation. Another key requirement for effective implementation therefore relates to the development and use of effective indicators.

#### Appropriate use of value and sensitive valuation methods

Conceptual presentations of the notion of value, and its relationship to well-being, varied greatly between frameworks from those which offer no consideration at all (F3UES, GSaH, GIEH) to those which employ a primarily economic basis of assessment (ecosystem service cascade, VNN framework) and those which emphasize a combination of monetary and non-monetary approaches (IPBES). Interpretation and criticism of the notion of value was likewise diverse among workshop participants and the need to acknowledge both intrinsic and utilitarian approaches to valuing the natural environment was expressed by members of both groups. The notion of value both in the context of the discussion of conceptual models in workshop one and the discussion of definitions and individual concepts in workshop two provided the greatest degree of plurality of meaning of any theme under consideration in the knowledge-brokering process. A key outcome of workshop two (stressed by members of Group Two) centred on the appropriate assigning of monetary and non-monetary values to the natural environment and the use of community-focussed versus asset-based approaches to managing natural heritage. The challenge of acknowledging and understanding multiple interpretations and frames of reference around value, and the failure of many of the selected frameworks to meet this challenge, was therefore a key finding of the knowledge co-production process.

The IPBES approach, however, was unique among the identified frameworks in its accounting for value. Whilst incorporating the provision of ecosystem services, it sees the latter as one of a variety of mediating factors in the fulfilment of a good life (Diaz et al. 2015). Moreover, by also stressing the relevance of an intrinsic view of nature’s worth, it provides the only conceptual presentation inclusive of utilitarian, intrinsic and relational values. Central to the idea of relational value is a sense of kinship with natural processes and landscapes which speaks more clearly to that which motivates people to identify with and conserve the natural environment. Indeed, the IPBES approach is the only framework of those identified that gives explicit consideration to the value of heritage as a mediating cultural concern in the assessment of health outcomes related to GI (whereas most others included some acknowledgement of social or cultural interaction *within* green spaces there was no evidence of cultural associations *with* the natural environment itself). It could be claimed that, within frameworks which adopt an ecosystem services approach, the notion of cultural ecosystem services encompasses an appreciation of heritage value, though explicit reference to this is not made in the supporting material. However, the use of ecosystem services, if based on a monetary valuation approach, can be at odds with participatory valuation approaches which tend to emphasise a plurality of values, many of which are incommensurate with economic models. The IPBES framework provided the only direct acknowledgement of the importance of taking account of relational and cultural value in an assessment of human well-being and, as such, provides a promising starting point for navigating polarised stances related to nature valuation (see Mansur et al. 2022 and section “Opportunities for further integration and effective implementation of the principles for leveraging GI as a public health intervention”).

Another key concern which emerged through the outputs of workshop one was the absence of emphasis in the identified frameworks on understanding value (and well-being) through participatory processes. This reflects assertions elsewhere in the social-ecological literature that the planning of GI, and conservation efforts more generally, has been shown to have greater efficacy when adopting a user-centred approach (Biggs et al. 2012). Although most of the frameworks, as anthropocentric approaches to environmental assessment and planning, take human well-being as their primary goal, there is overall little emphasis on the widening of participation towards achieving this goal. The integration of user-participation as a formal consideration in the process of value creation and associated well-being outcomes appears to be another challenge not met by the frameworks identified in this study. Again, an emphasis on ecosystem service provision is conspicuous with only one of the ES-centric models studied here (the MGIP framework) acknowledging the benefit of such approaches in capturing value.

#### Issues of scale in exploring GI benefits to human health and well-being

Scale, both geographical and temporal, was highlighted in the knowledge-brokering process as a consideration which can influence the presence and impact of benefits to human well-being derived from GI as observed, for example, by Coutts and Hahn (2015) in the delivery of vital ecosystem services from urban GI. Those conceptual frameworks with a strong foundation in ecology gave the most comprehensive account of the relevance of geographical scale in the treatment of concepts related to ecosystem function and connections with human exposure and health and well-being outcomes. For example, the F3UES takes a spatial approach to mapping stocks and flows of ecosystem services, acknowledging that such processes, and their effective assessment, are scale-dependent. Likewise, within the MGIP and Ecosystem Services Cascade models, there is special consideration given to the idea that the various ecosystem functions associated with multi-functional green space influence people at different scales (e.g. local, distant, uni-directional) and Tzoulas et al. (2007) acknowledge the effect of scale and resolution in assessing a range of indicators. However, though frameworks with a GI focus considered scale in their conceptualization, actual examples tended to be regional-to-global. Conversely, frameworks grounded in public health disciplines, though drawing on evidence collected at a range of scales, failed to address explicitly the role of scale itself. A focus on scale-effects directly related to health outcomes was therefore lacking from all frameworks and an understanding of the scales at which GI-related processes directly impact health and well-being requires further clarification.

### Opportunities for further integration and effective implementation of the principles for leveraging GI as a public health intervention

Fully meeting these multiple challenges related to scale, value and complexity may require a process-oriented approach based on inter- and trans-disciplinary work and an associated suite of tools and methods. In particular, this will require expertise from the natural, health and social sciences as well as arts and heritage researchers and practitioners. For instance, taking the issue of scale, ecosystem processes and societal preferences may be enacted and felt at different scales. Here, disciplines such as Geographical Information Science and landscape ecology expertise may be needed for mapping ecological processes (e.g. Haase et al. 2014; Dennis et al. 2018) in addition to social and arts practice methods (e.g. participatory action research, Ashton et al. 2021) to capture the complex relationship between GI, valuation processes and health and well-being impacts. In order to articulate these processes and relationships, and make them more accessible to a range of participants, the use of effective boundary objects to bridge between disciplines, users and practitioners may provide a potential pathway. For example, the use of social-ecological traits (Andersson et al. 2021) provides a promising route to linking social and ecological processes at different scales in urban environments and represents a heuristic through which key ideas related to GI, ageing, health and well-being could be communicated.

Workshop participants highlighted the need for robust indicators (particularly of health and well-being and GI quality) for which secondary “big data” and social-media derived datasets are proving effective in the study of urban GI use (e.g. Ilieva and McPhearson 2018). Such sources of information could be considered in support of principles six (relevance of scale), seven (adopting a comparative approach) and eight (identifying mechanisms) to identify, for example, the scale at which GI influences behaviour and space-use, the spatial variation in such use and the behaviours which are associated with well-being outcomes (Coutts and Hahn 2015).

In terms of pathways to communicating and implementing outcomes and a plurality of values that may arise from the application of our proposed principles, recent work that places emphasis on relational value and visioning (e.g. Mansur et al. 2022) provides a promising way forward. Combining different perspectives on the value of, and cultural ties with, urban GI, as exemplified by the Urban Nature Futures Framework (IPBES Expert Group on Scenarios and Models, Ibid.) may provide a useful framing of urban GI planning with which our principles could be combined. For example, carrying out visioning work (historically a top-down enterprise) in a participatory setting with a range of stakeholders (principle four) to identify motivations, preferences and values towards urban nature (principle five) for different demographic groups (principles two and seven) has the potential to address key concerns identified in the knowledge-brokering process. These include the possibility of transcending long-debated, difficult dichotomies such as those around instrumental versus intrinsic value. Such an approach could be enhanced and fine-tuned by ensuring that user perspectives are captured across scales and locations (principles one, six and seven), for example, by using available methods such as Q-methodology to capture diverse views and value plurality (Watts and Stenner 2005). Additionally, examples exist of open-source democratic valuation (http://maps.humanities.manchester.ac.uk/ghia-web/value) and data-visualisation (http://maps.humanities.manchester.ac.uk/ghia-web/extract) tools that capture spatial variation in GI and its assigned value (relating to principles four, six and seven), and which have been delivered through inter-disciplinary research. Such tools can increase the reach and accessibility of key methods, helping to overcome practical and logistical barriers to trans-disciplinary work. Ultimately, these approaches should work towards the democratization of decision-making around GI through, for example, the promotion of active citizenship (sensu Buijs et al. 2016) governance approaches.

In addition to the use of appropriate methods and techniques to operationalise the principles, their application for the leveraging of GI benefits as a public health intervention should also be considered with respect to the following general guidance.

1. They should be applied sensitively, considering unique social-ecological contexts, where particular natural, cultural, socio-economic and political landscapes may dictate a variety of needs and interpretation when promoting human health and well-being. For example, at different times and locations, socio-cultural factors (Principle 7) may limit or moderate the influence of GI on health outcomes (Principle 8).
2. The scale of operation (Principle 6) is of crucial importance when carrying out research or intervention-based work. Acknowledgement of this, and the scale of operation itself, will dictate the format in which and degree to which participatory (Principle 3) and user-centred (Principle 1) approaches can be carried out. Similarly, the modes with which individuals and communities relate to and value their local or regional environment (Principle 4) will be influenced by their relative position in terms of their geographic (Principle 7) and temporal (Principle 2) contexts.
3. In order to account for such diversity, a flexible outlook (Principle 5) is necessary to effectively capture the narratives played out through such interactions. Likewise, the application of these principles in general should be done so in a flexible rather than prescriptive way which acknowledges that circumstances dictate the degree to which each one is emphasized. For example, research with a focus on environmental justice may employ a comparative approach to identifying inequalities in green and blue space provision (Principle 7) as a point of departure with a subsequent emphasis on stakeholder participation (Principle 3). Alternatively, epidemiological studies will necessitate clear hypotheses and theoretical stances in explorations of specific health-related outcomes (Principle 8) and long-term strategic GI planning should focus on dynamics across scales (Principle 6) and consider multiple interpretations of value towards effective provision of social-ecological benefits and avoidance of harms (Principle 4).

## Conclusion

The knowledge-brokering process identified a range of considerations and perspectives on GI, ageing health and well-being within both practice and research. The degree to which these are integrated into existing framings and how these shortcomings might be overcome were likewise facilitated by this form of co-production. The work presented here, both in terms of the principles themselves and the knowledge-brokering approach to their creation, can be adapted to other studies exploring the benefits of the natural environment to human health and well-being. We recommend that the co-production of knowledge be carried out in an iterative and context-aware manner such that insight gained at all stages of the research process can be cross referenced, using these nine principles as a road map to knowledge production. The process itself can act as a template for wider trans-disciplinary research and is readily transferable to a range of complex and interconnected topics where there are inter-dependencies between physical and social domains.

## Supplementary Information

Below is the link to the electronic supplementary material.

Supplementary file1 (PDF 268 kb)
