# Supplementary material for: Integrating knowledge on green infrastructure, health and well-being in ageing populations: Principles for research and practice
Source: Ambio. 2022 Aug 6;52(1):107–25. doi: 10.1007/s13280-022-01765-5 (PMC9666607; doi:10.1007/s13280-022-01765-5)
Supplement: Supplementary file 1 — Supplementary file1 (PDF 268 kb) [file 13280_2022_1765_MOESM1_ESM.pdf]

AMBIO

Electronic Supplementary Materials

This supplementary material has not been peer reviewed.

Title: **Integrating knowledge on green infrastructure, health and well-being in ageing populations: Principles for research and practice**

Authors: Matthew Dennis, Adam Barker, Jamie Anderson, Jenna Ashton, Gina Cavan, Penny Cook, David French, Anna Gilchrist, Philip James, Chris Phillipson, Konstantinos Tzoulas, Phil Wheater, Ada Wossink, Sarah Lindley.

Appendix S1

Identifying contemporary approaches to conceptualising the inter-relationship of green infrastructure, ageing, health and well-being.

An attempt to identify existing conceptual frameworks, through a search of the scientific literature, which addressed the dominant themes of green infrastructure, ageing, health and well-being was carried out within the Scopus search engine (September2016), combining the following three groups of search terms:

1 Ecosystem functions/green infrastructure

TITLE-ABS

(“greenspace” OR "green space" OR "green infrastructure" OR "urban nature" OR "urban biodiversity" OR "urban park" OR "city park" OR "public garden" OR "municipal garden" OR "botanic garden" OR "community garden" OR "city garden" OR allotment OR "urban planting" OR "urban landscaping" OR "common land" AND ("conceptual framework" OR "conceptual model")

2 Health & Wellbeing

TITLE-ABS

(“wellbeing” OR “wellness” OR "quality of life" OR "life satisfaction" OR "purpose in life" OR "personal growth" OR “dependence” OR “discrimination” OR “loneliness” OR “exclusion” OR “anxiety” OR "mental fatigue" OR "social support" OR "mental health" OR "psychological health" OR "health behaviour" OR "quality of life" OR "physical health" OR “disability” AND ("conceptual framework" OR "conceptual model")

3 Ageing

TITLE-ABS

(“senior” OR “elderly” OR "older people" OR "older persons" OR “ageing” OR “aging” AND ("conceptual framework" OR "conceptual model")
